# How Do Cells of the Oligodendrocyte Lineage Affect Neuronal Circuits to Influence Motor Function, Memory and Mood?

**DOI:** 10.3389/fncel.2018.00399

**Published:** 2018-11-16

**Authors:** Renee E. Pepper, Kimberley A. Pitman, Carlie L. Cullen, Kaylene M. Young

**Affiliations:** Menzies Institute for Medical Research, University of Tasmania, Hobart, TAS, Australia

**Keywords:** NG2 glia, myelin, oligodendrocyte, anxiety, motor function, depression, learning, neuronal activity

## Abstract

Oligodendrocyte progenitor cells (OPCs) are immature cells in the central nervous system (CNS) that can rapidly respond to changes within their environment by modulating their proliferation, motility and differentiation. OPCs differentiate into myelinating oligodendrocytes throughout life, and both cell types have been implicated in maintaining and modulating neuronal function to affect motor performance, cognition and emotional state. However, questions remain about the mechanisms employed by OPCs and oligodendrocytes to regulate circuit function, including whether OPCs can only influence circuits through their generation of new oligodendrocytes, or can play other regulatory roles within the CNS. In this review, we detail the molecular and cellular mechanisms that allow OPCs, newborn oligodendrocytes and pre-existing oligodendrocytes to regulate circuit function and ultimately influence behavioral outcomes.

## Introduction

Within the central nervous system (CNS), cells of the oligodendrocyte lineage are critical regulators of circuit function. Oligodendrocyte progenitor cells (OPCs) generate oligodendrocytes that elaborate myelin membrane to ensheath discrete axon segments, effectively reducing axonal capacitance and enabling the saltatory conduction of action potentials. However, their role in circuit regulation does not stop there. Herein, we detail the maturation of OPCs into pre-myelinating and myelinating oligodendrocytes and outline how each cell type can influence neural network construction, operation and plasticity to ensure healthy CNS function.

## Oligodendrocyte Progenitor Cells

### Origin and Other Sources of Heterogeneity

**Figure 1:**
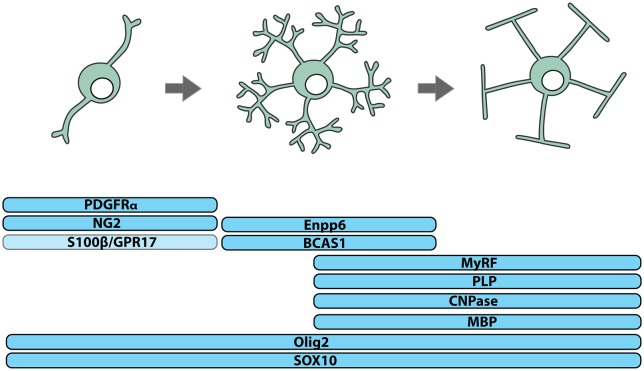
Identifying cells of the oligodendrocyte lineage. Cells of the oligodendrocyte lineage can be subdivided into three stages of differentiation based on protein expression: oligodendrocyte progenitor cells (OPCs), premyelinating oligodendrocytes and myelinating oligodendrocytes. Essentially all OPCs co-express the NG2 proteoglycan and PDGFRα, while only a subset express S100β and/or the G-protein coupled receptor, GPR17. Premyelinating oligodendrocytes express Breast Carcinoma Amplified Sequence-1 (BCAS1) and Ectonucleotide Pyrophosphatase Phosphodiesterase 6 (Enpp6), and upregulate Myelin Regulatory Factor (MyRF). Myelinating oligodendrocytes express myelin-related proteins including Myelin Basic Protein (MBP), Proteolipid Protein (PLP) and 2’,3’-Cyclic-nucleotide 3’-phosphodiesterase (CNPase). All cells of the oligodendrocyte lineage express the transcription factors OLIG2 and SOX10.

OPCs, also known as oligodendrocyte precursors or NG2-glia, can be identified by their expression of platelet-derived growth factor receptor α (PDGFRα; Stallcup and Beasley, 1987; Hart et al., 1989; Pringle et al., 1992; Rivers et al., 2008) or the NG2 proteoglycan (Zhu et al., 2008) and the transcription factors SOX10 (Kuhlbrodt et al., 1998) and OLIG2 (Lu et al., 2000; Zhou et al., 2000; Dimou et al., 2008; Figure 1). In human brain development, PDGFRα^+^ OPCs are detected in the forebrain at ~10 weeks of gestation and increase in number until ~15 weeks (Jakovcevski et al., 2009). A high density of OPCs in the ventricular and subventricular zones of the ganglionic eminence and cortex suggests that OPCs are generated by ventral and dorsal neural stem cell populations (Jakovcevski et al., 2009) and a stream of PDGFRα^+^ OPCs bridging the ganglionic eminence and cortex, suggests that OPCs of ventral origin migrate to populate the cortex (Rakic and Zecevic, 2003).

The mixed dorsal and ventral origin of cortical OPCs has been verified by histological (Ivanova et al., 2003) and cre-lox lineage tracing (Kessaris et al., 2006) studies of the developing mouse forebrain, which revealed that OPCs are first generated in the ventricular zone of the medial ganglionic eminence (MGE) at embryonic day (E)12 and migrate in a number of directions, including into the developing cortex, arriving by ~E16. OPCs are subsequently generated from the ventricular zones of the lateral ganglionic eminence (LGE) and cortex (Kessaris et al., 2006), and while MGE-derived OPCs do not persist postnatally, those derived from the LGE and cortex remain throughout adulthood (Kessaris et al., 2006).

OPCs in the spinal cord have a similarly mixed origin. In the human (Hajihosseini et al., 1996) and mouse (Fu et al., 2002; Masahira et al., 2006) spinal cord, the ventrally-located premotor neuron (pMN) domain is the first and major source of OPCs. However, studies of mouse development indicate that ~3 days after these OPCs are produced, others are generated from more dorsal domains (Cai et al., 2005; Fogarty et al., 2005; Vallstedt et al., 2005; Tripathi et al., 2011).

OPCs of different embryonic origin can have similar electrophysiological properties (Tripathi et al., 2011) and following the conditional ablation of OPCs from one site of origin, OPCs from another expand to occupy the unpopulated territory (Kessaris et al., 2006), indicating that a level of phenotypic and functional redundancy exists between different OPC populations. However, the long-term consequence of ablating OPCs from a given origin has not been explored, and it is unclear how origin contributes to reports of postnatal OPC phenotypic and functional heterogeneity. In the postnatal CNS, only some OPCs express S100β (Vives et al., 2003; Hachem et al., 2005) or the G protein-coupled receptor 17 (GPR17), and GPR17^+^ OPCs are less likely to differentiate to produce oligodendrocytes than GPR17-negative OPCs (Viganò et al., 2016; Figure 1). It is important to consider that such differences in gene expression and function could result from divergent signaling within the postnatal CNS, as adult human OPCs have been shown to locally upregulate the fibroblast growth factor receptor (FGFR)1 when they are associated with active demyelinating lesions (Clemente et al., 2011).

### OPCs Interact With the CNS Vasculature

Once generated, human and mouse OPCs associate with the vascular endothelium and migrate along and between blood vessels, extending a leading process prior to translocation of the cell body (Tsai et al., 2016). As OPCs migrate, they also divide, so that they expand in number to occupy the CNS (van Heyningen et al., 2001; Kelenis et al., 2018). Postnatally, OPCs continue to proliferate (Rivers et al., 2008; Psachoulia et al., 2009; Zhu et al., 2011; Clarke et al., 2012; Hughes et al., 2013). In acute brain slices generated from early postnatal mice, OPCs divide asymmetrically to produce an OPC and a new oligodendrocyte or symmetrically to produce two OPCs or two oligodendrocytes (Zhu et al., 2011). Symmetric and asymmetric OPC divisions also occur in the adult mouse brain, however, *in vivo* imaging has revealed that OPCs occupy and maintain spatially discrete domains through a process of self-repulsion and that proliferation rarely immediately precedes differentiation—rather OPC differentiation appears to trigger the proliferation of adjacent OPCs, ensuring homeostatic progenitor cell replacement (Hughes et al., 2013).

**Figure 2:**
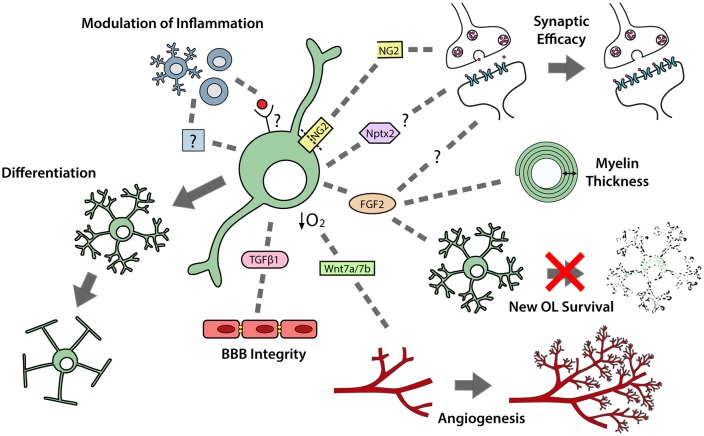
OPCs perform multiple functions in the developing and adult central nervous system (CNS). OPCs differentiate to produce premyelinating and myelinating oligodendrocytes. OPCs also secrete a number of paracrine factors that can regulate neuroinflammation; synaptic efficacy; myelin thickness; premyelinating oligodendrocyte survival; angiogenesis and blood brain barrier (BBB) integrity.

In development, while using the vasculature as a scaffold for migration, OPCs exert a strong regulatory influence over angiogenesis and vascular cell function (Figure 2). OPCs secrete transforming growth factor (TGF)β1 to promote tight junction protein expression by endothelial cells, which enhances the integrity of the blood brain barrier (BBB; Seo et al., 2014). They also secrete unidentified factors that enhance the proliferation of endothelial cells (Yuen et al., 2014) and pericytes (Maki et al., 2015). Furthermore, as OPCs expand to occupy the brain, they enter regions that have insufficient vasculature to meet oxygen demands. Hypoxia activates the oxygen-sensing subunits of the hypoxia-inducible factor (HIF) complex within OPCs and drives their secretion of Wnt7a/7b, to promote angiogenesis and increase oxygen supply to that region of the developing brain (Yuen et al., 2014; Figure 2).

### OPCs Modulate Neuroinflammation

In postnatal development, microglia are important regulators of OPC maintenance and oligodendrogenesis, as their pharmacological depletion results in fewer OPCs populating the corpus callosum and impaired oligodendrogenesis in the corpus callosum, cerebellum and cortex (Hagemeyer et al., 2017). This effect may, in part, result from a loss of microglial-derived transglutaminase-2 (Tgm2), as *Tgm2* knockout mice also have reduced OPC proliferation in the corpus callosum and the conditional deletion of *Tgm2* from microglia produces a small but significant decrease in the number of callosal oligodendrocytes produced by P28 (Giera et al., 2018). OPCs can reciprocally regulate microglial function, as the transgenic ablation of NG2^+^ cells from the adult rat brain activates microglia (Figure 2), leading to hippocampal neuronal cell death (Nakano et al., 2017).

The extent to which OPCs interact with peripheral immune cells in the healthy CNS is unclear, however their expression of genes associated with antigen presentation and inflammation (Zhang et al., 2014) suggests that they can modulate or even exacerbate neuroinflammation (Figure 2). Data obtained using an adoptive transfer model of experimental autoimmune encephalomyelitis (EAE) support this idea, as they show that OPCs can respond to activated Th17 cells (Wang et al., 2017). Th17 cells are known to secrete the cytokine interleukin-17 (IL-17), which can bind to IL-17 receptors expressed by OPCs to activate notch1 signaling and a pro-inflammatory cascade that leads to immune-mediated demyelination of the CNS (Wang et al., 2017).

### OPCs Modulate Synaptic Efficacy

Postnatally, cortical OPCs play an important role in memory formation, as the activity-dependent cleavage of NG2 can modulate α-amino-3-hydroxy-5-methyl-4-isoxazolepropionic acid (AMPA) and *N*-methyl-D-aspartate (NMDA) receptor mediated currents, and enhance NMDA receptor-dependent long-term potentiation in pyramidal neurons (Sakry et al., 2014; Figure 2). As OPCs are found throughout the CNS, it is possible that they influence synaptic strength in other regions via cleaved NG2 or the secretion of other factors able to modulate neuronal communication and synaptic plasticity (reviewed by Parolisi and Boda, 2018). For example, Nptx2 (neuronal pentraxin 2), also known as neuronal activity regulated pentraxin (Narp), is a secreted immediate-early gene product that can modulate the clustering of AMPA receptors (O’Brien et al., 1999), regulate excitatory synapse function (Gu et al., 2013; Pelkey et al., 2015), and influence the functional integration of interneurons into neural circuits (Pelkey et al., 2015). As developmental OPCs express Nptx2 (Sakry et al., 2015), Nptx2 secretion may be another mechanism by which OPCs influence synaptic efficacy (Figure 2).

## Pre-Myelinating Oligodendrocytes

While OPCs perform a number of functions in the CNS, their best-known function is the life-long generation of oligodendrocytes (Figures 1, 2). OPCs differentiate to produce oligodendrocytes during postnatal development (Zhu et al., 2008, 2011) and throughout adulthood (Dimou et al., 2008; Rivers et al., 2008; Kang et al., 2010; Zhu et al., 2011; Young et al., 2013). They initially differentiate into pre-myelinating oligodendrocytes, retaining their expression of SOX10 and OLIG2, losing PDGFRα and NG2, and gaining expression of Breast Carcinoma Amplified Sequence 1 (BCAS1; Zhang et al., 2014; Fard et al., 2017), Ectonucleotide Pyrophosphatase Phosphodiesterase 6 (ENPP6; Zhang et al., 2014; Xiao et al., 2016) and Myelin Regulator Factor (MyRF; Cahoy et al., 2008; Emery et al., 2009; Figure 1). This early differentiation step also involves significant morphological change, most obviously the symmetric elaboration of a dense network of fine processes (Trapp et al., 1997).

In the P7–P21 rat cortex ~20% of pre-myelinating oligodendrocytes are degenerating at any one time (Trapp et al., 1997) and ~78% die within 2 days of differentiation in the adult mouse cortex (Hughes et al., 2018). The survival of pre-myelinating oligodendrocytes is enhanced by FGFR signaling *in vitro* (Palser et al., 2009) and β1-integrin (Benninger et al., 2006) or glutamatergic (Kougioumtzidou et al., 2017) signaling *in vivo*. While it is possible that this largely transient cell population performs currently unknown regulatory functions in the CNS, its only known function is to act as a reservoir of cells available for further differentiation into mature, myelinating oligodendrocytes.

## Myelinating Oligodendrocytes

### Myelination

**Figure 3:**
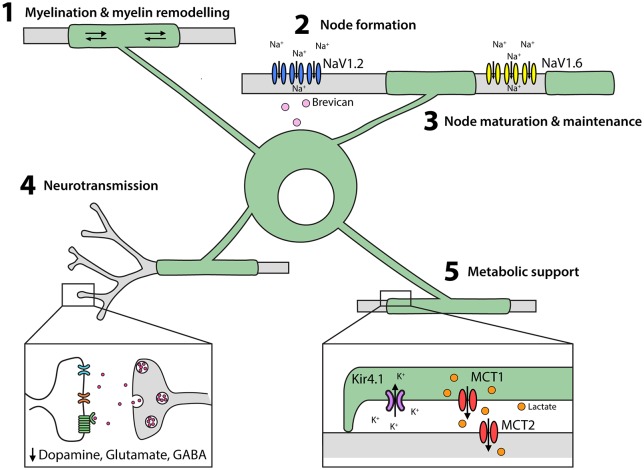
Oligodendrocytes perform multiple functions in the developing and adult CNS. (1) Oligodendrocytes elaborate and remodel myelin internodes. (2) Oligodendrocytes secrete extracellular matrix molecules, such as brevican, which trigger the clustering of NaV1.2 into pre-nodes. Myelination is also important for nodal maturation (NaV1.2 is exchanged for NaV1.6) and nodal maintenance. (3) Oligodendrocytes and their myelin modulate neuronal excitability and neurotransmitter release. (4) Oligodendrocytes provide lactate to axons via the periaxonal space and remove K^+^ ions.

The major function of oligodendrocytes is to add myelin internodes to both excitatory (Young et al., 2013; Tomassy et al., 2014) and inhibitory (Micheva et al., 2016; Stedehouder and Kushner, 2017; Stedehouder et al., 2017, 2018) neurons in the CNS (Figure 3). Oligodendrocyte maturation requires the transcription factor MyRF (Emery et al., 2009), but is influenced by extrinsic signals, including neuronal activity (Barres and Raff, 1999; Lundgaard et al., 2013; Gibson et al., 2014). At the onset of myelination, oligodendrocytes extend motile processes to contact axons (Kirby et al., 2006; Hughes et al., 2013). Following contact, the oligodendrocyte process flattens to form a sheet that is tightly attached at the cytoplasmic surfaces. The leading edge of this growing myelin sheath spirals around the axon, extending the new wrap underneath the preceding one, while simultaneously extending laterally along the axon (Snaidero et al., 2014). Myelin initiation requires Arp2/3 complex-dependent actin assembly (Zuchero et al., 2015) and ADF/cofilin-1-dependent actin depolymerization drives myelin wrapping (Nawaz et al., 2015). The major myelin protein, myelin basic protein (MBP), is also necessary for actin disassembly and myelin compaction, while 2’, 3’-Cyclic-nucleotide 3’-phosphodiesterase (CNPase) counteracts myelin compaction allowing myelin formation, as well as the formation of uncompacted myelinic channels within the sheath (Snaidero et al., 2017).

A single oligodendrocyte elaborates and supports myelin sheaths on numerous axons. Time-lapse imaging of the developing zebrafish spinal cord has revealed that oligodendrocytes initially over-produce short myelin sheaths (Czopka et al., 2013; Hines et al., 2015; Mensch et al., 2015) and that sheath retraction and extension is regulated by local calcium signaling induced by neuronal activity (Baraban et al., 2018; Krasnow et al., 2018). Long-duration, high-amplitude calcium bursts facilitate calpain-mediated sheath retraction, while lower-amplitude, short-duration calcium busts correlate with the rate of sheath extension (Baraban et al., 2018; Krasnow et al., 2018). Sheath retraction, stabilization and extension occurs within a 5-h window (Czopka et al., 2013) and while final internode length is influenced by intrinsic properties of the maturing oligodendrocyte, it can also be influenced by extrinsic factors such as axon diameter (Bechler et al., 2015). Extrinsic factors also regulate myelin thickness, for example, activation of FGFR2 in the paranodal loops of the adult mouse spinal cord, enhances MyRF expression and increases Extracellular Signal-Related Kinase (ERK)1/2 and, in turn, mammalian Target of Rapamycin (mTOR)C1 activity to increase myelin thickness (Furusho et al., 2017).

### Myelinating Oligodendrocyte Survival

Once formed, oligodendrocytes are long-lived cells (Yeung et al., 2014; Tripathi et al., 2017; Hill et al., 2018; Hughes et al., 2018). In mice, the overall density of CC1^+^ oligodendrocytes was found to increase in the corpus callosum, motor cortex and spinal cord throughout adulthood, but the density of GFP^+^ oligodendrocytes born prior to P60 (*Opalin-CreER^T2^ :: Tau-mGFP* mice) remained unchanged until old age (≥P240; (Tripathi et al., 2017). Consistent with these data, the *in vivo* imaging of oligodendrocytes in the somatosensory cortex of adult mice, revealed that mature oligodendrocytes are remarkably stable (Hill et al., 2018; Hughes et al., 2018). However, these experiments also revealed that adult-born oligodendrocytes add new myelin internodes to partially myelinated axons throughout life, and that this addition is not accompanied by the loss of pre-existing internodes (Hill et al., 2018), suggesting that adult oligodendrogenesis is not simply required for cell replacement or myelin turnover, but instead represents a novel form of neural plasticity, that modifies the myelination of existing circuits.

### Myelin Remodeling

Axons are not uniformly myelinated along their length in the adult mouse brain—some are unmyelinated, at least for long stretches, and others are partially myelinated (Tomassy et al., 2014). The myelin profile of individual axons can be modified by the addition of new internodes or by modifying existing internodes in a process termed myelin remodeling. When new oligodendrocytes are added to the adult mouse somatosensory cortex, they elaborate an equivalent number of internodes, that are similar in length to those produced by developmentally-born oligodendrocytes in the same region (Hughes et al., 2018). However, the myelin internodes retain a certain level of plasticity after elaboration (Hill et al., 2018). For a single oligodendrocyte, some sheaths remain stable over time while others change in length (Figure 3), suggesting that internode plasticity is modulated at the level of the internode, rather than the level of the oligodendrocyte. Overall, ~81% of sheathes are stable, ~15% extend and ~4% retract between P60 and P90 (Hill et al., 2018) and this drops to ~1% of sheaths extending or retracting between P300 and P420 (Hughes et al., 2018), suggesting that myelin remodeling decreases with ageing. As internode extension would require uncoupling of the paranodal junction and either stretching of the existing myelin sheath or the addition of new myelin membrane, it is difficult to imagine how this might occur. It is also unclear what regulates this subtle form of myelin remodeling, or the purpose of this plasticity for axon function. However, it conceivably provides a mechanism for the fine-tuning of myelination after internode elaboration is complete.

The process of myelin remodeling differs between the mouse optic nerve and cortex, perhaps because optic nerve axons are essentially fully myelinated during development. In the optic nerve, adult-born oligodendrocytes morphologically differ from those generated in the same region during postnatal development (Young et al., 2013). Oligodendrocytes born between 4 and 6 months of age elaborate more internodes that are significantly shorter than those elaborated in development (Butt et al., 1994; Young et al., 2013). Additionally, the number of new oligodendrocytes added over this period exceeds the number that would be required to myelinate the small number of unmyelinated or partially myelinated optic nerve axons remaining (Young et al., 2013). These data may suggest that oligodendrocytes are less stable in the optic nerve compared to the cortex, as oligodendrocyte and myelin turnover could explain the need for so many additional oligodendrocytes. Alternatively, developmentally-born oligodendrocytes may significantly remodel their sheaths, to provide space for adult-born oligodendrocytes to elaborate short myelin sheaths. The addition of numerous short myelin sheaths by adult-born oligodendrocytes is predicted to modify axonal conduction velocity (Young et al., 2013), but may also ensure that a larger number of oligodendrocytes are available to meet the metabolic needs of axons (see below).

### The Generation and Maintenance of Nodes of Ranvier

While the dendrites and soma of neurons receive input, the axon supports output i.e., action potential initiation and propagation. The myelination of axons reduces their effective membrane capacitance and speeds up action potential conduction (Rasminsky and Sears, 1972), with action potentials being regenerated at the small (~1 μm) excitable axon domains called nodes of Ranvier. Nodes of Ranvier are characterized by a high density of voltage-gated sodium channels (NaV) and are immediately flanked by the paranodal and juxtaparanodal domains of myelin internodes. In the CNS, clustering of NaV at the nodes of Ranvier is coordinated by oligodendrocytes (reviewed by Freeman et al., 2016; Zhang and Rasband, 2016).

In the early stages of node formation, the NaV subunits NaV1.1 and NaV1.2 cluster to form pre-nodes, and this process can be triggered in cultured retinal ganglion and hippocampal neurons by exposure to oligodendrocyte-conditioned medium (Kaplan et al., 2001; Freeman et al., 2015). *In vivo*, it has been shown that glial-derived extracellular matrix molecules, such as brevican, regulate NaV clustering in an ankyrin G-dependent manor (Feinberg et al., 2010; Freeman et al., 2016; Figure 3), with ankyrin-G bridging Neurofascin 186 in the axonal cytoskeleton and NaV in the axonal membrane (Wang et al., 2014; Xu and Cooper, 2015). Node formation along cortical projection neurons is concomitant with myelination, however pre-nodes containing NaV1.2 form along excitatory axons in the developing rat optic nerve (Kaplan et al., 2001) and on GABAergic axons in the rat and mouse cortex before the onset of myelination (Freeman et al., 2015). Cell-attached patch clamp recordings from cultured hippocampal interneurons support the ability of these pre-nodes to increase conduction velocity (Freeman et al., 2015), however, myelination is required for nodal maturation and the maintenance of saltatory conduction.

During nodal maturation NaV1.2 is replaced with NaV1.6 (Figure 3). While oligodendrocyte-conditioned medium is insufficient to induce the clustering of NaV1.6, this process does occur in neurons that are co-cultured with astrocytes and oligodendrocytes (Freeman et al., 2015). During myelination, the axonal proteins contactin and contactin-associated protein (caspr) interact with neurofascin 155 in the oligodendrocyte myelin loops (Peles et al., 1997; Bhat et al., 2001; Charles et al., 2002; Sherman et al., 2005) to form the paranodal junctions that stabilize the nodes. Disruption of the paranodal junctions by gene deletion (Suzuki et al., 2004) or demyelination (Hamada and Kole, 2015) has significant effects on sodium channel (particularly NaV1.6) expression and clustering, and NaV and voltage-gated potassium channels (KV) diffuse between the node and the paranode, short-circuiting the node (Rosenbluth, 2009). These data indicate that oligodendrocytes play a role in node of Ranvier formation, maturation and maintenance (Figure 3).

### Myelinating Oligodendrocytes Regulate Neuronal Excitability

It has been suggested that myelination can affect the intrinsic excitability of axons, as spontaneous supra-threshold depolarizations and antidromic action potentials are ectopically generated in the distal regions of demyelinated axons (Hamada and Kole, 2015). This may relate to the ability of oligodendrocytes to regulate potassium homeostasis in highly myelinated white matter regions (Larson et al., 2018). In mice, the conditional deletion of the inwardly rectifying potassium channel, Kir4.1, from mature oligodendrocytes, does not alter myelination but slows potassium clearance in the corpus callosum and optic nerve, causing pathological neuronal hyperexcitability (Larson et al., 2018).

Oligodendrocytes and their associated myelin may also influence neurotransmitter release and efficacy in the CNS (Figure 3), as mice with reduced oligodendrocyte number and/or impaired myelination have enhanced evoked dopamine release in the striatum (Roy et al., 2007) and elevated dopamine expression in the prefrontal cortex (Xu et al., 2010). Furthermore, in mice with altered myelin ultrastructure, glutamate and glycine expression is increased in the superior olivary complex and GABA expression increased in the amygdala and ventral hippocampus (Maheras et al., 2018). These findings may reflect the ability of myelination to reduce neuronal excitability, but may alternatively be a secondary effect of myelin loss, whereby neurons undergo pre- or post-synaptic modifications in an attempt to compensate for impaired regulation of the circuit.

### Myelination Regulates Conduction Velocity and Synchronicity

Myelination not only allows the rapid arrival of action potentials, but coordinates action potential synchrony (Freeman et al., 2016). It has been suggested that for large-diameter myelinated axons, such as motor-neurons, in which action potentials can travel at 80 ms^−1^, myelination may principally enable speed, however, in cortical neurons where conduction is much slower (~0.5–4.5 ms^−1^), myelination may principally promote synchrony (Freeman et al., 2016). Cortical neurons generate action potentials in an oscillatory rhythm, which synchronizes their discharge with high precision (Gray et al., 1989) and the maturation of neural synchrony across adolescence is associated with the development of cognitive functions, including working memory and executive processes (James et al., 2008). GABAergic interneurons, particularly parvalbumin (PV)^+^ basket cells, are critical for establishing neural synchrony, and while a single interneuron may be sufficient to synchronize the firing of multiple pyramidal neurons, when coupled to other interneurons, via gap junctions, they can precisely synchronize the oscillations of many pyramidal neurons within the network (reviewed Uhlhaas et al., 2009). Oligodendrocytes and their associated myelin may facilitate the long-range synchronization of different cortical regions by ensuring the precision and frequency of the neural oscillations (Uhlhaas et al., 2009).

Oligodendrocytes are known to coordinate action potential arrival times and allow neurons to fire at high frequencies. Following the demyelination of layer V cortical pyramidal neurons, action potential conduction is no longer saltatory, but instead propagates as a slow, broad, continuous wave, and fails at high firing frequencies (Hamada et al., 2017). In systems such as the auditory system, the fidelity of timing of action potential arrival is critical, and in mice lacking CNS expression of *claudin11*, in which the passive properties of compact CNS myelin are altered, the myelinated, small diameter axons of the auditory pathway have slowed conduction and the resulting temporal dispersion (loss of synchronicity) is predicted to distort auditory perception (Maheras et al., 2018). Furthermore, dysmyelination of auditory system neurons is associated with spike failure and action potential “jitters” (Kim et al., 2013).

For myelination to precisely regulate action potential arrival time across multiple axons in any circuit, internodes must either: (i) be laid down with incredible precision during ensheathment; (ii) retain some level of plasticity, to allow adjustments to be made in internode length after ensheathment, or (iii) possess the ability to modify their ultrastructure in order to regulate conduction. While there is evidence that internode length can be adjusted (Hill et al., 2018; Hughes et al., 2018), the level of remodeling reported in the cortex appears insufficient to achieve synchronicity. However, node of Ranvier length may also be modulated, and on a larger scale, to fine-tune conduction velocity (Ford et al., 2015; Arancibia-Cárcamo et al., 2017). This has been difficult to reconcile experimentally as both the conditional deletion of a cohesion regulatory protein, *Esco2*, from all cells of the oligodendrocyte lineage (Schneider et al., 2016), and the overexpression of Anosmin-1 in mouse development (Murcia-Belmonte et al., 2016), lengthen nodes of Ranvier, but have opposing effects on action potential conduction velocity in the corpus callosum. Such differences may be explained by associated changes in internode length, axon diameter, myelin thickness, NaV1.6 density or axonal metabolic support, but highlight the need for carefully designed experiments that can selectively examine the contribution of nodal plasticity to conduction velocity regulation. While the mechanisms that underpin conduction velocity tuning are not fully elucidated, oligodendrocyte depolarization has been shown to directly increase conduction velocity (Yamazaki et al., 2007, 2014).

### Oligodendrocytes Provide Metabolic Support to Axons

Oligodendrocytes provide metabolic support to axons, allowing them to influence neuronal homeostasis independently of conduction velocity modulation (Figure 3). It is for this reason, that mice lacking proteolipid protein (PLP), that have compact, though unstable myelin, are initially able to sustain conduction, but ultimately experience axon degeneration (Klugmann et al., 1997; Griffiths et al., 1998). Similarly, mice lacking CNPase have normal appearing myelin, but elaborate internodes that have abnormal inner tongue processes and paranodal loops (Lappe-Siefke et al., 2003; Rasband et al., 2005; Edgar et al., 2009), and, in the case of large caliber axons, have disrupted myelinic channels—a phenotype sufficient to cause progressive axonal degeneration (Zuchero et al., 2015). By contrast, oligodendrocytes in mice lacking MBP produce thin, uncompacted myelin sheaths that are insufficient to support saltatory conduction, but largely prevent axon degeneration (Loers et al., 2004). By comparing the phenotypes of these mice, it is clear that oligodendrocytes play an important role in supporting neuron survival, and that the elements of the myelin sheath that are critical for supporting action potential conduction, differ from those required for neuronal survival.

Oligodendrocytes require cytoplasmic myelinic channels to transfer short carbon-chain energy metabolites, such as pyruvate and lactate, to axons (reviewed Philips and Rothstein, 2017; Figure 3). Neuronal activity is associated with glutamate release, which binds NMDA receptors on oligodendrocytes and increases their glucose uptake and lactate production (Saab et al., 2016). Oligodendrocytes express the monocarboxylate transporter (MCT)1 which transports monocarboxylate metabolites and has a high affinity for lactate transport (Lee et al., 2012). As oligodendrocytes accumulate intracellular lactate it is transported via MCT1 into the periaxonal space and taken into neurons by MCT2 (Fünfschilling et al., 2012; Lee et al., 2012). Consistent with this mechanism, the conditional deletion of *Mct1* from oligodendrocytes results in severe axonal injury and motor neuron death in mice (Lee et al., 2012). Furthermore, in brain slices, MCT1- and MCT2-deficiency result in axonal degeneration, but only MCT1-deficiency can be rescued by the exogenous application of L-Lactate (Suzuki et al., 2011; Lee et al., 2012), as MCT2 can transport lactate directly into the axon.

Within the axon, lactate is converted to pyruvate that enters the mitochondrial citric acid cycle to drive oxidative phosphorylation and the generation of ATP, which is necessary to maintain the activity of NaV and KV to sustain the continuous, repetitive firing of action potentials (Almeida et al., 2001; Saez et al., 2014). Indeed, the failure of de/dys-myelinated axons to fire at high firing frequencies (Kim et al., 2013; Hamada et al., 2017), may be explained by the loss of oligodendrocyte-derived metabolic support. In this way, the lactate-shuttle overcomes the limited ability of axons to meet their own energy demands and is a critical role fulfilled by oligodendrocytes and myelin across a number of circuits (Figure 3).

## Oligodendrocyte Lineage Cells Affect Motor Circuit Function

Multiple sclerosis (MS) is an autoimmune and neurodegenerative disease in which central demyelination and axonal loss are associated with significant motor impairment (reviewed by Trapp and Nave, 2008) and changes in motor function and coordination are frequently used as behavioral indicators of the onset of demyelination in preclinical models of MS, particularly in the EAE model (Tripathi et al., 2010; Moore et al., 2013; Grace et al., 2017). The requirement of myelination for normal motor circuit function is also highlighted by the motor phenotype that develops in *Plp1*-null mice, in which subtle changes in myelin structure slow action potential conduction in the brain (Gould et al., 2018) and spinal cord (Klugmann et al., 1997; Petit et al., 2014). A detailed behavioral analysis of these mice revealed that gross motor coordination on the rotorod test was unaffected, but that fine motor coordination was disrupted, as evidenced by gait abnormalities, uncoordinated and slower swimming, extended time to complete a puzzle box and reduced marble burying by 3 months of age, as well as reduced swimming distance and less digging time by 9 months of age (Gould et al., 2018). Furthermore, ablation of the oligodendrocyte-specific transcription factor *Myrf* from all cells of the oligodendrocyte lineage or from oligodendrocytes disrupts central myelination and severely impairs motor performance in the rotorod test (Koenning et al., 2012; McKenzie et al., 2014). While these phenotypes may directly result from myelin dysfunction in the CNS, they could alternatively be a secondary consequence of altered microglial or astrocytic function, as the pharmacological depletion of microglia from *Cnp1* knockout mice relieves their catatonia, suggesting that, at least in this instance, motor dysfunction was not the direct result of myelin abnormalities (Janova et al., 2018).

While developmental myelination is required for normal motor function, it is not yet clear whether motor function is influenced by ongoing adult myelination. Preventing the formation of new myelinating oligodendrocytes in adulthood, by conditionally deleting* Myrf* from adult OPCs, does not disrupt existing myelination or elicit detectable dysfunction in rotorod performance, but does impair coordinated motor performance, as evidenced by a reduced running speed on the complex running wheel (McKenzie et al., 2014). By contrast, the conditional deletion of *Esco2*, from all Sox10^+^ cells, to induce apoptosis of the proliferating OPCs, produces a severe deficit in motor coordination, that can be detected in the beam crossing and grid walk tests after 6 weeks (Schneider et al., 2016). In this model, the ablated OPCs were primarily GPR17-negative OPCs, and a compensatory increase in the proliferation of un-recombined GPR17^+^ OPCs meant that OPC number was equivalent between control and gene-deleted mice. However, the number of newborn (BrdU-labeled) oligodendrocytes added to the white matter was effectively halved, the nodes of Ranvier and paranodes lengthened and the conduction velocity of callosal axons slowed (Schneider et al., 2016). While this phenotype may be the result of reduced oligodendrogenesis, it may also partially reflect a change in OPC composition, which is not a feature of the *Myrf*-deletion model. Further research is needed to fully understand the role that adult OPCs and ongoing myelination play in the regulation of motor function.

## Oligodendrocyte Lineage Cells Affect Learning and Cognition

As more than half of all people with MS experience cognitive decline (reviewed; Rocca et al., 2015), it is likely that myelination also exerts a significant influence on cognitive circuits. Cognitive impairment can be an early feature of this disease, as ~20% of people with early MS or clinically isolated syndrome fail four or more neuropsychological assessment tasks, indicating significant impairment in attention, executive function and learning and memory (Baysal Kiraç et al., 2014). The development of cognitive impairment temporally correlates with demyelination of gray matter regions, including the neocortex, particularly the cingulate cortex, thalamus, hippocampus, cerebellum and spinal cord (Geurts and Barkhof, 2008) and cortical lesion load and cortical volume independently correlate with the level of cognitive impairment (Calabrese et al., 2009).

The idea that myelination is critical for normal cognitive function is also supported by rodent preclinical models that preferentially induce oligodendrocyte loss and demyelination of the corpus callosum (Xu et al., 2010), hippocampus (Xu et al., 2017) or medial prefrontal cortex (Yang et al., 2017) and impair working memory. Social isolation during development, which has no effect on oligodendrocyte number, but results in thinner myelin in the prefrontal cortex and hippocampus, also impairs working memory (Makinodan et al., 2012; Cao et al., 2017). As increasing the thickness of already elaborated myelin sheaths conversely facilitates contextual fear memory acquisition (Jeffries et al., 2016), myelin sheath thickness appears to be an important regulator of circuits relevant to cognition.

It is possible that learning not only requires developmental but adult myelination, as a strong association exists between learning and oligodendrogenesis. Indeed, training mice on a complex running wheel is associated with a rapid increase in the number of pre-myelinating oligodendrocytes present in the motor cortex and subcortical white matter (Xiao et al., 2016), and environmental enrichment, somatosensory enrichment or skilled reaching training increase the number of newly differentiated oligodendrocytes in brain regions relevant to each activity (Keiner et al., 2017; Hughes et al., 2018). In rodents, learning the skilled reaching task is also associated with an increase in fractional anisotropy of the white matter region underlying the somatosensory cortex contralateral to the trained forepaw (Sampaio-Baptista et al., 2013), and in humans, learning to juggle is similarly associated with an increase in fractional anisotropy of the white matter underlying the right posterior intraparietal sulcus (Scholz et al., 2009). In both cases, magnetic resonance imaging detected changes in fractional anisotropy in brain regions activated by the task, and in rodents, the increased fractional anisotropy correlated with an increase in MBP expression in the same brain region (Sampaio-Baptista et al., 2013). These data suggest that the learning-induced changes detected by magnetic resonance imaging reflect myelin addition or changes in existing myelin, rather than altered axon caliber or branching.

Despite oligodendrocyte generation occurring alongside learning, few studies have examined the requirement of oligodendrogenesis and/or myelination for learning and memory functions. To address this question, McKenzie et al. (2014) used a cre-lox transgenic approach to conditionally delete *Myrf* from OPCs in the adult mouse brain. When *Myrf*-deleted mice were placed on the complex running wheel and running speed used as a surrogate for motor learning, *Myrf*-deleted mice performed worse than control mice at all time-points examined (McKenzie et al., 2014; Xiao et al., 2016). As this effect is seen within the first few hours of carrying out the learning task (Xiao et al., 2016), efficient motor learning may require the rapid production of new oligodendrocytes, but it is unclear whether pre-myelinating and/or myelinating oligodendrocytes are required. If premyelinating cells alone are required, they may be providing paracrine support to the network, however if myelinating cells are required, the new myelin may modify conduction velocity or support the increased metabolic load placed on the circuit.

## Oligodendrocyte Lineage Cells Influence Emotional State

Perturbations that result in reduced central myelination can result in the development of mood disorders such as anxiety and depression. In humans this is largely correlative, with white matter abnormalities being well documented in psychiatric disorders associated with social withdrawal and anxiety (reviewed by Parnanzone et al., 2017), and demyelinating disorders often being accompanied by co-morbid depression (reviewed by Arnett et al., 2008). Similarly, in rodents, focal demyelination of the medial prefrontal cortex (Yang et al., 2017), diffuse white matter injury (van Tilborg et al., 2018) and EAE are all associated with increased anxiety- and depressive-like behaviors. Changes in myelin may contribute to this phenotype, however it is also possible that altered OPC function is a consequence of demyelination and contributes to the development of an anxiety-like phenotype. OPCs and astrocytes are known to produce interleukin 33 in the brain (Zhang et al., 2014), and the performance of *interleukin 33* knockout mice in the elevated plus maze and open field test is indicative of reduced anxiety (Dohi et al., 2017), suggesting that increased interleukin 33 release could conversely increase anxiety. The medial prefrontal cortex, amygdala and ventral hippocampus of *interleukin-33* knockout mice also contain more cfos^+^ neurons (Dohi et al., 2017), which may reflect increased neuronal activity, however as this is a constitutive knockout, it is unclear whether interleukin-33 affects the development or function of the circuit.

Supporting the idea that OPCs can influence anxiety, the focal genetic ablation of OPCs from the prefrontal cortex of young, adult *NG2-Cre :: iDTR* transgenic mice was sufficient to produce anxiety-like behaviors within 7 days (Birey et al., 2015). While this phenotype may result from impaired local oligodendrogenesis, knocking down FGF2 expression in prefrontal cortical OPCs recapitulates the anxiety-like phenotype (Birey et al., 2015), suggesting that FGF2 release from OPCs is a critical regulator of circuit function in this region. Glutamate uptake by astrocytes is also reduced following focal OPC ablation and the response of pyramidal neurons to glutamatergic input is impaired, as fewer GluR1-containing AMPA receptors are expressed in the membrane (Birey et al., 2015). Curiously, mice lacking OPCs only in the prefrontal cortex did not exhibit any signs of anhedonia, but ablating OPCs from the entire CNS was associated with reduced pleasure seeking in the sucrose preference test (Birey et al., 2015), suggesting that OPC function in other brain regions has a greater impact on depressive-like phenotypes.

Neonatal maternal separation, early weaning and chronic variable stress-paradigms also impair oligodendrogenesis and myelination of the medial prefrontal cortex, and produce anxiety- and depressive-like symptoms (Kodama et al., 2008; Ono et al., 2008; Yang et al., 2017; Liu et al., 2018). It has been shown that neonatal maternal separation stress reduces HDAC1/2 expression which impairs oligodendrogenesis, and that blocking HDAC1/2 recapitulates the phenotype (Yang et al., 2017). However, the mechanism by which stress induces anxiety- and depressive-like behaviors is likely to be complex, as stressed mice have narrower nodes of Ranvier and paranodes in the corpus callosum (Miyata et al., 2016), an increased number of PV^+^ interneurons in the prefrontal cortex, fewer cfos^+^ neurons in the prefrontal cortex (Shepard et al., 2016), and the stress hormone corticosterone can change the function of hippocampal neural stem/progenitor cells, by directing their generation of oligodendrocytes (Chetty et al., 2014). However, a role for impaired oligodendrogenesis in mediating this phenotype is further supported by genetic approaches that impair oligodendrogenesis and myelination and similarly produce an anxiety-like phenotype.

Oligodendrocyte number and myelination is impaired following the oligodendrocyte-specific knockdown of ErbB (Roy et al., 2007) and the oligodendrocyte-specific deletion of *Olig2* (Chen et al., 2015). In each case, mice showed impaired movement and increased anxiety-like behavior in the open field and elevated plus maze (Roy et al., 2007; Chen et al., 2015). This behavioral change was also associated with altered neurotransmitter release. ErbB knockdown increased evoked dopamine release in the striatum (Roy et al., 2007), while *Olig2* knockout increased glutamate expression in the cortical gray matter and increased the density of glutamatergic vesicles at synaptic terminals (Chen et al., 2015), suggesting that oligodendrocyte loss may precipitate an anxiety-like phenotype by dysregulating neurotransmitter signaling in the CNS. By contrast, mice that lack *claudin11* in the CNS have perturbed myelination that is accompanied by an increase in glutamate and glutamine expression in the superior olivary complex and an increase in GABA expression in the amygdala and ventral hippocampus, and show reduced anxiety-like behavior (Maheras et al., 2018), suggesting that impaired myelination can have opposing effects depending on the brain regions affected.

## Closing Remarks

We have sufficient evidence to conclude that cells of the oligodendrocyte lineage influence motor coordination, cognition and emotional state. While the behavioral outcomes are very different in each case, it is likely that common mechanisms of circuit modification are responsible. Key mechanisms include paracrine signaling by cells of the oligodendrocyte lineage as well as conduction velocity modulation and the provision of metabolic support by myelinating oligodendrocytes. However, more research is required to fully understand how OPCs, premyelinating and myelinating oligodendrocytes contribute to brain plasticity and enable neuronal circuits to be regulated and remain adaptable to experience throughout life.

## Author Contributions

KY, RP, CC and KP wrote the article.

## Conflict of Interest Statement

The authors declare that the research was conducted in the absence of any commercial or financial relationships that could be construed as a potential conflict of interest.
